# Transcriptomic analysis of *Streptomyces clavuligerus ΔccaR::tsr:* effects of the cephamycin C-clavulanic acid cluster regulator CcaR on global regulation*

**DOI:** 10.1111/1751-7915.12109

**Published:** 2014-01-22

**Authors:** R Álvarez-Álvarez, A Rodríguez-García, I Santamarta, R Pérez-Redondo, A Prieto-Domínguez, Y Martínez-Burgo, P Liras

**Affiliations:** 1 Área de Microbiología, Departamento de Biología Molecular, Facultad de CC, Biológicas y Ambientales, Universidad de LeónLeón, Spain; 2 Instituto de Biotecnología de Léon (INBIOTEC), Parque Científico de LeónLeón, Spain

## Abstract

*Streptomyces clavuligerus* ATCC 27064 and *S. clavuligerus* Δ*ccaR::tsr* cultures were grown in asparagine-starch medium, and samples were taken in the exponential and stationary growth phases. Transcriptomic analysis showed that the expression of 186 genes was altered in the *ccaR*-deleted mutant. These genes belong to the cephamycin C gene cluster, clavulanic acid gene cluster, clavams, holomycin, differentiation, carbon, nitrogen, amino acids or phosphate metabolism and energy production. All the clavulanic acid biosynthesis genes showed M_c_ values in the order of −4.23. The *blip* gene-encoding a β-lactamase inhibitory protein was also controlled by the cephamycin C-clavulanic acid cluster regulator (M_c_ −2.54). The expression of the cephamycin C biosynthesis genes was greatly reduced in the mutant (M_c_ values up to −7.1), while the genes involved in putative β-lactam resistance were less affected (M_c_ average −0.88). Genes for holomycin biosynthesis were upregulated. In addition, the lack of clavulanic acid and cephamycin production negatively affected the expression of genes for the clavulanic acid precursor arginine and of miscellaneous genes involved in nitrogen metabolism (*amtB*, *glnB*, *glnA3*, *glnA2*, *glnA1*). The transcriptomic results were validated by quantative reverse transcription polymerase chain reaction and luciferase assay of *luxAB*-coupled promoters. Transcriptomic analysis of the homologous genes of *S. coelicolor* validated the results obtained for *S. clavuligerus* primary metabolism genes.

## Introduction

*Streptomyces* species are the largest group of antibiotic-producing micro-organisms. Antibiotic biosynthesis genes are usually clustered and their expression is regulated in response to nutritional environment, culture time and cell density. Most of the gene clusters for antibiotic biosynthesis contain regulators responding to these parameters (Martín and Liras, 2010). Regulators of LysR-(Pérez-Redondo *et al*., 1998) or large ATP-binding regulators of the LuxR family (LAL)-type (Wilson *et al*., 2001; Antón *et al*., 2004; Tahlan *et al*., 2007) are present in several clusters, but the most common ones are the activator proteins belonging to the SARP (*Streptomyces* antibiotic regulatory proteins) family. They are present in many antibiotic clusters (Bate *et al*., 2002; Garg and Parry, 2010; He *et al*., 2010) and bind specific deoxyribonucleic acid sequences activating expression of antibiotic biosynthesis genes.

Cephamycin C biosynthesis in *Streptomyces cattleya* is controlled by a SARP-type protein encoded by *thnU*, a gene that is not located in the cephamycin C cluster (Rodríguez *et al*., 2008). In *Streptomyces clavuligerus*, a similar protein, cephamycin C-clavulanic acid cluster regulator (CcaR), encoded by a gene located in the cephamycin C cluster, controls both cephamycin and clavulanic acid (CA) biosynthesis (Pérez-Llarena *et al*., 1997; Santamarta *et al*., 2011). The two clusters are located side by side in the chromosome.

Specific sequences for the binding of SARP proteins have been described in antibiotic biosynthesis genes (Wietzorrek and Bibb, 1997). In *S. clavuligerus*, triple heptameric conserved sequences are responsible for CcaR binding and controls the expression of the *lat, cefF, cefD* and *ccaR* genes in the cephamycin C cluster. In the CA cluster, CcaR binds sequences upstream of *ceaS2*, which encodes the first enzyme of the CA pathway, and of *claR*, for the LysR-type regulator controlling late steps in CA biosynthesis (Santamarta *et al*., 2011).

At the beginning of this study, the *S. clavuligerus* genome was not published, but many *S. clavuligerus* genes had been deposited in the database and additional genes were available through the DSM (Delft, The Netherlands) *S. clavuligerus* sequencing project. Therefore, we constructed microarrays containing probes for 800 genes of the *S. clavuligerus* genome as well as for 7728 genes of the *Streptomyces coelicolor* genome in order to compare the housekeeping genes of both strains. This microarray has been used to detect gene expression in different *S. clavuligerus* mutants. In this article, we report the results observed for *S. clavuligerus ΔccaR::tsr*, a strain lacking the SARP regulator that controls cephamycin C and CA biosynthesis.

## Results

### Transcriptomic analysis of antibiotic biosynthesis genes in *S. clavuligerus* ATCC 27064 and *S. clavuligerus* Δ*ccaR::tsr*

**Figure 1:**
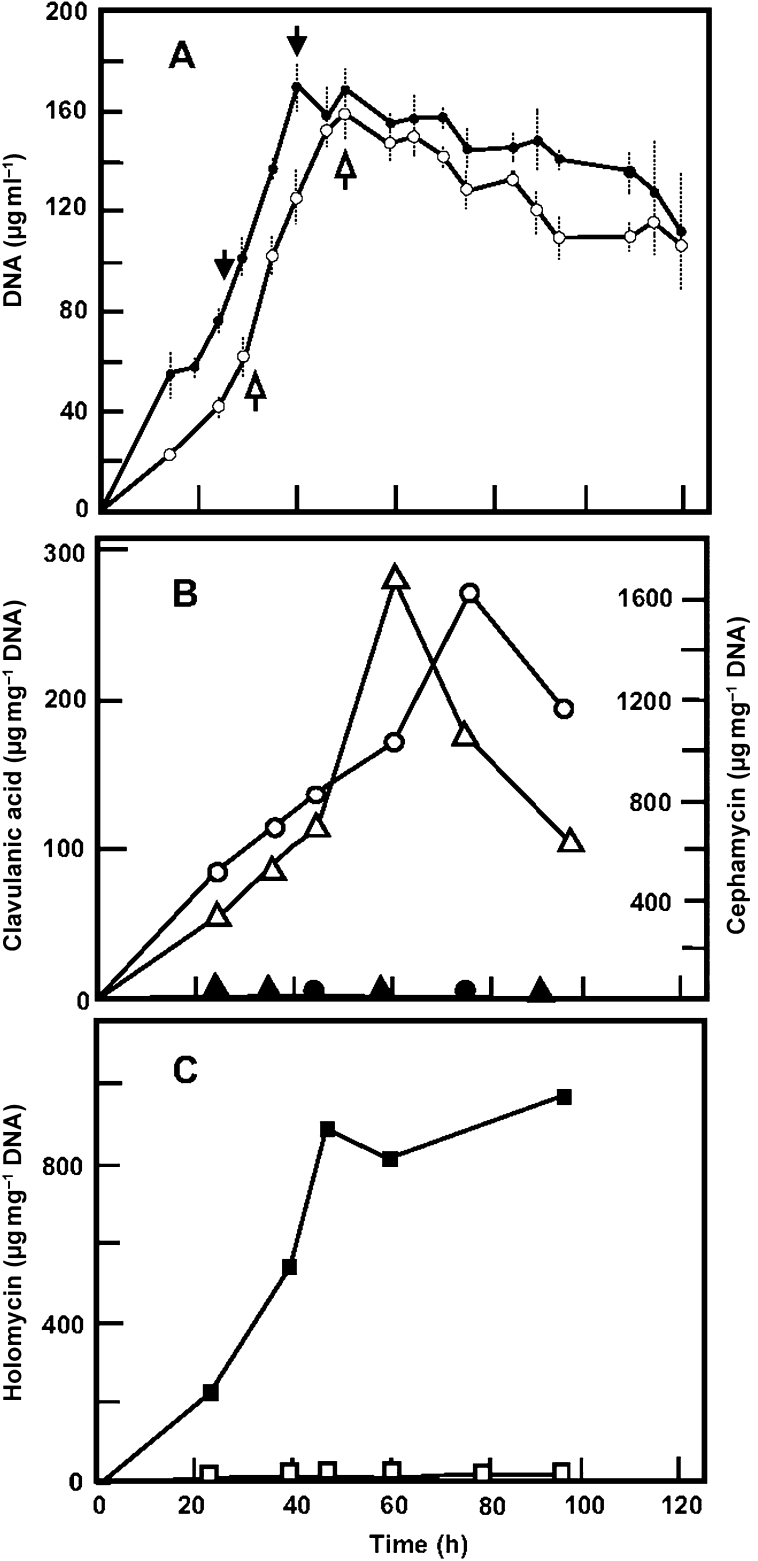
Growth and antibiotic production in Asparagine-starch medium. Strains used: *S. clavuligerus* ATCC 27064 (open symbols) and *S. clavuligerus ΔccaR::tsr* (closed symbols). A. Growth (circles) as measured by the DNA content. B. Cephamycin C (circles) or clavulanic acid (triangles) production. C. Holomycin production (squares).

*Streptomyces clavuligerus* ATCC 27064 and *S. clavuligerus* Δ*ccaR::tsr* cultures were grown in asparagine-starch (SA) medium, and samples were taken in the exponential and stationary growth phases (Fig. 1) as indicated in Experimental Procedures. The microarrays analysis showed differences in expression in 186 genes of the *ccaR*-negative mutant at one specific sampling time at least (Table 1).

**Table 1:** *Streptomyces clavuligerus* Δ*ccaR::tsr* gene expression as compared with *S. clavuligerus* ATCC 27064

			Exponential phase		Stationary phase	
Code	Gene	Product	Mc	FDR	Mc	FDR
Clavulanic acid biosynthesis						
SCLAV_4189	*cyp*	Cytochrome P450	−5.34	< 1E^−06^	−5.19	< 1E^−06^
SCLAV_4191	*claR*	Transcriptional regulator	−4.61	< 1E^−06^	−3.99	< 1E^−06^
SCLAV_4187	*orf12*	Beta-lactamase protein-like	−5.09	< 1E^−06^	−4.51	< 1E^−06^
SCLAV_4181	*gcas*	Carboxylase	−5.44	< 1E^−06^	−4.98	< 1E^−06^
SCLAV_4186	*orf13*	Export pump	−3.73	< 1E^−06^	−3.25	< 1E^−06^
SCLAV_4185	*orf14*	Acetyl transferase	−4.77	< 1E^−06^	−3.84	< 1E^−06^
SCLAV_4183	*oppA2*	Oligopeptide-binding protein	−5.28	< 1E^−06^	−4.72	< 1E^−06^
SCLAV_4182	*orf16*	DUF482 domain-containing protein	−5.03	< 1E^−06^	−4.40	< 1E^−06^
SCLAV_4180	*orf18*	Penicillin-binding protein	−0.49	7.48E^−04^	−0.58	2.02E^−05^
SCLAV_4179	*orf19*	Penicillin-binding protein	−0.61	9.36E^−03^	−0.65	1.99E^−03^
SCLAV_4178	*orf20*	Cytochrome P450	−0.95	3.93E^−05^	−1.68	< 1E^−06^
SCLAV_4196	*bls2*	Beta-lactam synthetase 2	−6.38	< 1E^−06^	−5.36	< 1E^−06^
SCLAV_4190	*car*	Clavaldehyde dehydrogenase	−3.94	< 1E^−06^	−3.03	< 1E^−06^
SCLAV_4194	*cas2*	Clavaminato sintase 2	−6.55	< 1E^−06^	−6.00	< 1E^−06^
SCLAV_4197	*ceaS2*	Carboxyethyl arginine synthetase 1	−5.78	< 1E^−06^	−5.33	< 1E^−06^
SCLAV_4193	*oat2*	Similar to ornithine acetyltransferase	−2.67	< 1E^−06^	−2.81	< 1E^−06^
SCLAV_4192	*oppA1*	Oligopeptide-binding protein	−4.97	< 1E^−06^	−4.44	< 1E^−06^
SCLAV_4195	*pah2*	Proclavaminate amidinohydrolase 2	−6.66	< 1E^−06^	−6.01	< 1E^−06^
Cephamycin C biosynthesis						
SCLAV_4198	*pcbR*	Beta-lactam antibiotics resistance	−1.02	4.88E^−05^	−0.92	9.76E^−05^
SCLAV_4199	*pcbC*	Isopenicillin N synthetase	−3.25	< 1E^−06^	−2.82	< 1E^−06^
SCLAV_4200	*pcbAB*	ACV Synthetase	−5.21	< 1E^−06^	−3.85	< 1E^−06^
SCLAV_4201	*lat*	L-lysine epsilon amino transferase	−7.19	< 1E^−06^	−5.87	< 1E^−06^
SCLAV_4202	*blp*	Similar to beta-lactamase inhibitory protein	−6.23	< 1E^−06^	−4.84	< 1E^−06^
SCLAV_4203	*orf10*	Secreted protein	−3.40	< 1E^−06^	−3.20	< 1E^−06^
SCLAV_4204	*ccaR*	Transcriptional regulator	−7.12	< 1E^−06^	−7.48	< 1E^−06^
SCLAV_4205	*cmcH*	Cabamoyl transferase	−5.96	< 1E^−06^	−4.16	< 1E^−06^
SCLAV_4206	*cefF*	Deacetyl cephalosporin C synthetase	−6.83	< 1E^−06^	−5.48	< 1E^−06^
SCLAV_4207	*cmcJ*	Methyl transferase	−7.23	< 1E^−06^	−5.32	< 1E^−06^
SCLAV_4208	*cmcI*	Cepahalosporin hydroxylase	−7.48	< 1E^−06^	−6.25	< 1E^−06^
SCLAV_4210	*cefD*	Isopenicillin N epimerase	−6.15	< 1E^−06^	−5.18	< 1E^−06^
SCLAV_4211	*cefE*	Deacetoxycephalosporin C synthetase (DAOCS)	−5.12	< 1E^−06^	−4.45	< 1E^−06^
SCLAV_4212	*pcd*	Piperideine carboxylate dehydrogenase	−3.40	< 1E^−06^	−2.08	< 1E^−06^
SCLAV_4213	*cmcT*	Efflux protein	−3.74	< 1E^−06^	−2.29	< 1E^−06^
SCLAV_4214	*pbp*	Penicillin-binding protein	−0.51	2.25E^−03^	−0.63	4.27E^−05^
Proteins *blip*						
SCLAV_4456	*atpA*	Similar to ABC transporter ATP-binding domain	−1.08	3.95E^−02^	−1.62	2.88E^−04^
SCLAV_4457	*atpA2*	Similar to ABC transporter ATP-binding domain	−1.07	< 1E^−06^	−1.65	< 1E^−06^
SCLAV_4455	*blip*	Beta-lactamase inhibitory protein	−2.54	< 1E^−06^	−2.19	< 1E^−06^
SCLAV_4452		Putative regulatory protein	−0.71	2.32E^−01^	−0.99	2.71E^−02^
SCLAV_4453		Hypothetical protein	−0.76	1.10E^−03^	−0.98	< 1E^−06^
Clavams biosynthesis						
SCLAV_p1072	*orf8*	Serine hydroxymethyltransferase	−2.59	< 1E^−06^	−3.18	< 1E^−06^
SCLAV_p1076	*pah1*	Proclavaminate amidinohydrolase 1	−0.98	3.99E^−02^	−3.23	< 1E^−06^
SCLAV_p1077	*oat1*	Ornithine acetyltransferase isoenzyme	−1.58	6.48E^−04^	−2.60	< 1E^−06^
SCLAV_p1078	*cvm6P*	Pyridoxal phosphate dependent aminotransferase	−1.04	9.64E^−03^	−2.28	< 1E^−06^
SCLAV_p1079	*cvm7P*	Transcriptional regulator	−0.90	1.23E^−03^	−1.97	< 1E^−06^
SCLAV_2922	*cvm6*	Pyridoxal phosphate dependent aminotransferase	−0.61	1.61E^−02^	−1.98	< 1E^−06^
SCLAV_2923	*cvm5*	Flavin-dependent oxidoreductase	−1.51	2.27E^−05^	−2.88	< 1E^−06^
SCLAV_2924	*cvm4*	Deacetylcephalosporin C acetyltransferase	−1.69	7.88E^−03^	−2.56	< 1E^−06^
SCLAV_2925	*cas1*	Clavaminate synthase 1	−3.32	2.79E^−02^	−4.29	1.01E^−03^
SCLAV_2926	*cvm1*	Aldo/keto reductase family 2	−1.17	1.80E^−04^	−1.99	< 1E^−06^
SCLAV_2927	*cvm2*	Ribulose 5 phosphate epimerase	−1.14	2.09E^−05^	−2.40	< 1E^−06^
SCLAV_2928	*cvm3*	Oxidoreductase	−0.99	1.30E^−03^	−2.18	< 1E^−06^
SCLAV_2932	*cvm13*	Asparaginase	−0.95	7.30E^−04^	−1.45	< 1E^−06^
Holomycin biosynthesis						
SCLAV_5267	*hlmA*	Acetyl transferase	5.95	< 1E^−06^	4.41	< 1E^−06^
SCLAV_5268	*hlmB*	Acyl-CoA dehydrogenase	6.03	< 1E^−06^	4.60	< 1E^−06^
SCLAV_5269	*hlmC*	Thioesterase	6.11	< 1E^−06^	4.39	< 1E^−06^
SCLAV_5270	*hlmD*	Probable dehydrogenase	6.11	< 1E^−06^	4.07	< 1E^−06^
SCLAV_5272	*hlmF*	DNA/pantothenate metabolism flavoprotein	5.47	< 1E^−06^	3.83	< 1E^−06^
SCLAV_5273	*hlmG*	Hypothetical protein – *Frankia sp.* EAN1pec	5.41	< 1E^−06^	3.69	< 1E^−06^
SCLAV_5275	*hlmI*	Putative reductase – *S. coelicolor*	3.96	< 1E^−06^	4.23	< 1E^−06^
SCLAV_5278	*hlmM*	Putative transcriptional regulator	2.01	2.59E^−04^	1.36	8.01E^−03^
Arginine biosynthesis						
SCLAV_0799	*argB*	Acetylglutamate kinase	−2.15	< 1E^−06^	−2.98	< 1E^−06^
SCLAV_0801	*argC*	N-acetyl-gamma-glutamyl-phosphate reductase	−1.70	3.58E^−02^	^−^2.49	3.43E^−04^
SCLAV_0798	*argD*	Acetonitrile aminotransferase	−1.67	3.19E^−03^	−2.60	< 1E^−06^
SCLAV_0796	*argG*	Argininosuccinate synthase	−2.39	< 1E^−06^	−2.91	< 1E^−06^
SCLAV_0795	*argH*	Argininosuccinate lyase	−2.37	1.29E^−05^	−2.81	< 1E^−06^
SCLAV_0800	*argJ*	Glutamate N-acetyltransferase	−2.32	2.99E^−05^	−3.32	< 1E^−06^
SCLAV_0797	*argR*	Transcriptional regulator	−1.45	1.30E^−04^	−2.34	< 1E^−06^
Drugs resistance						
SCLAV_0793	*epeA*	Transmembrane-transport protein	−0.19	8.42E^−01^	−1.59	2.42E^−03^
SCLAV_0794	*epeR*	TetR-family transcriptional regulator	−0.48	2.76E^−01^	−1.93	< 1E^−06^
Strict response						
SCLAV_0744	*relA*	ppGpp synthetase	1.19	5.69E^−04^	1.22	1.36E^−04^
Cell differentiation						
SCLAV_5713	*rarE*	Putative cytochrome P450	−0.72	5.18E^−03^	−1.22	< 1E^−06^
SCLAV_1816	*rarB*	RarB Roadblock/LC7 protein	−0.91	2.60E^−05^	−0.56	6.25E^−03^
SCLAV_1817	*rarC*	RarC protein	−0.90	< 1E^−06^	−0.51	4.58E^−03^
SCLAV_1818	*rarD*	RarD ATP/GTP-binding protein	−0.82	2.70E^−03^	−0.74	2.61E^−03^
Energy						
SCLAV_0790		Putative glycerophosphoryl diester phosphodiesterase	−0.91	1.16E^−03^	−1.29	< 1E^−06^
SCLAV_1370		Probable cytochrome c oxidase polypeptide IV	−0.82	9.14E^−05^	−0.15	4.78E^−01^
SCLAV_1372		Probable cytochrome c oxidase polypeptide II	−0.53	3.40E^−02^	−0.05	8.30E^−01^
SCLAV_1613	*aceE*	Pyruvate dehydrogenase E1 component	−0.54	1.32E^−04^	−0.95	9.70E^−12^
SCLAV_3564	*nuoA1*	NADH-quinone oxidoreductase chain	−0.96	< 1E^−06^	−0.48	8.03E^−03^
SCLAV_3970		Putative succinate dehydrogenase flavoprotein subunit	−1.83	< 1E^−06^	−0.95	4.30E^−04^
SCLAV_3969		Fumarate reductase iron-sulfur subunit	−1.67	1.82E^−05^	−1.15	1.66E^−03^
SCLAV_4767		Monophosphatase	−1.86	< 1E^−06^	−0.34	2.16E^−01^
Carbon metabolism						
SCLAV_0631	*glpF2*	Putative glycerol uptake facilitator protein	−1.49	1.12E^−05^	−1.95	< 1E^−06^
SCLAV_0632	*glpK2*	Putative glycerol kinase	−1.13	9.59E^−03^	−1.82	< 1E^−06^
SCLAV_0876	*gylR*	Glycerol operon regulatory protein	−0.31	3.62E^−01^	−1.34	< 1E^−06^
SCLAV_0877	*glpF1*	Putative glycerol uptake facilitator protein	0.30	6.19E^−01^	−0.42	2.76E^−01^
SCLAV_0878	*glpK1*	Glycerol kinase	−0.07	9.08E^−01^	−1.19	1.99E^−05^
SCLAV_0879	*glpD*	Glycerol-3-phosphate dehydrogenase	−0.02	9.35E^−01^	−0.94	< 1E^−06^
SCLAV_5509	*gap2*	Glyceraldehyde-3-phosphate dehydrogenase 2	−2.61	< 1E^−06^	−1.75	< 1E^−06^
SCLAV_4529	*glcP*	Glucose permease	1.79	< 1E^−06^	−0.60	7.97E^−02^
SCLAV_p0975		Ribulose-phosphate 3-epimerase	−1.25	< 1E^−06^	−1.36	< 1E^−06^
Nitrogen metabolism						
SCLAV_4534	*amtB*	Ammonium transporter	−4.10	< 1E^−06^	−5.04	< 1E^−06^
SCLAV_4535	*glnB*	Putative nitrogen regulatory protein P-II	−4.39	< 1E^−06^	−5.09	< 1E^−06^
SCLAV_p1452	*glnIII*	Glutamine synthetase III	−0.60	3.33E^−02^	−1.54	< 1E^−06^
SCLAV_1431	*glnA3*	Glutamine synthetase (Glutamate-ammonia ligase)	−4.67	< 1E^−06^	−5.32	< 1E^−06^
SCLAV_1416	*glnA2*	Glutamine synthetase I (Glutamate-ammonia ligase I)	−3.56	< 1E^−06^	−2.95	< 1E^−06^
SCLAV_1473		Glutamine synthetase	−0.88	6.84E^−03^	−2.35	< 1E^−06^
SCLAV_4660	*gluD1*	Glutamate transporter permease	−0.84	1.62E^−02^	−1.28	4.14E^−05^
SCLAV_0834	*glnA1*	Putative glutamine synthetase	−1.77	< 1E^−06^	−2.41	< 1E^−06^
Phosphate metabolism						
SCLAV_1719	*phoH*	Phosphate starvation-induced protein	1.25	1.37E^−04^	−0.06	8.67E^−01^
SCLAV_3166	*pstB*	PstB protein – Phosphate import ATP-binding	−0.93	1.41E^−02^	−1.50	1.14E^−05^
SCLAV_3167	*pstA*	PstA protein – Permease component	−1.12	4.84E^−05^	−1.61	< 1E^−06^
SCLAV_3168	*pstC*	PstC protein – Permease component	−1.11	2.41E^−02^	−1.77	4.12E^−05^
SCLAV_3169	*pstS*	PstS protein precursor – Periplasmic component	−0.93	3.10E^−02^	−1.41	1.66E^−04^
SCLAV_3220	*phoU*	Putative phosphate transport system regulatory protein	−1.01	3.52E^−04^	−1.23	< 1E^−06^
Lipid metabolism						
SCLAV_4986		Putative acetyl-coenzyme A synthetase	−0.55	4.01E^−02^	−2.18	< 1E^−06^
SCLAV_3406		Putative acetyl/propionyl CoA carboxylase alpha	−1.49	2.87E^−03^	−2.15	< 1E^−06^
SCLAV_3405		Putative acetyl/propionyl CoA carboxylase beta	−1.64	< 1E^−06^	−2.41	< 1E^−06^
Transcriptional and regulatory proteins						
SCLAV_p0826		Putative AraC-family transcriptional regulator	0.88	2.87E^−05^	−0.05	8.17E^−01^
SCLAV_p0894	*brp*	Gamma-butyrolactone receptor protein	0.86	7.97E^−04^	−0.55	1.98E^−02^
SCLAV_p1319		Putative transcriptional regulator AraC family	−1.72	1.75E^−04^	−1.10	1.03E^−02^
SCLAV_1096		Transcriptional regulator, GntR-family protein	−0.66	1.20E^−04^	−0.09	6.16E^−01^
SCLAV_1433		Putative regulatory protein	−1.18	2.33E^−02^	−0.60	1.94E^−01^
SCLAV_1621		Putative MerR-family transcriptional regulator	0.67	2.33E^−02^	−0.93	2.92E^−04^
SCLAV_1957	*adpA*	AraC-family transcriptional regulator	−1.22	5.42E^−04^	−1.43	1.52E^−05^
SCLAV_1958	*ornA*	Oligoribonuclease	−1.00	< 1E^−06^	−0.97	< 1E^−06^
SCLAV_2732		Two-component transcriptional regulator	1.31	2.20E^−03^	−0.23	6.00E^−01^
SCLAV_3001		Putative gntR-family transcriptional regulator	−0.68	8.78E^−04^	−1.51	< 1E^−06^
SCLAV_4054		WhiB-family transcriptional regulator	2.62	< 1E^−06^	0.32	3.13E^−01^
SCLAV_4937		Putative regulatory protein	1.05	9.79E^−06^	−1.06	< 1E^−06^
SCLAV_5278		AmphRI-like transcriptional regulator	2.01	2.59E^−04^	1.36	8.01E^−03^
Unknown function						
SCLAV_0018		Cytochrome P450 monooxygenase	−1.16	8.47E^−03^	−1.47	1.77E^−04^
SCLAV_0633		ATP–GTP-binding protein	−0.78	4.44E^−02^	−0.52	1.19E^−01^
SCLAV_0636		Putative large secreted protein	−0.76	1.59E^−02^	−0.17	5.91E^−01^
SCLAV_0646		Putative inhibitor of KinA	−0.93	3.99E^−02^	0.08	8.53E^−01^
SCLAV_0743		Peroxidase	1.46	< 1E^−06^	1.40	< 1E^−06^
SCLAV_1335		Two-component system sensor kinase	0.51	3.31E^−02^	−0.37	7.66E^−02^
SCLAV_1344		Conserved phosphoesterase	−0.78	1.36E^−02^	−1.11	8.41E^−05^
SCLAV_1564		Acetyl-CoA acetyltransferase	−2.06	< 1E^−06^	−2.68	< 1E^−06^
SCLAV_1565		Cytochrome P450 hydroxylase	−1.26	< 1E^−06^	−2.67	< 1E^−06^
SCLAV_1617		Hypothetical protein	1.02	< 1E^−06^	0.03	8.67E^−01^
SCLAV_1748		DUF143 domain-containing protein	0.45	1.28E^−02^	0.14	4.26E^−01^
SCLAV_1959		Sensor protein	−0.91	< 1E^−0^6	−1.36	< 1E^−06^
SCLAV_2623		SclavP3 predicted orf	1.03	1.10E^−02^	0.05	9.07E^−01^
SCLAV_2625		Subtilase-type protease inhibitor precursor	0.96	4.44E^−02^	−0.04	9.33E^−01^
SCLAV_3194		DUF1416 domain-containing protein	−0.77	2.64E^−02^	0.55	7.31E^−02^
SCLAV_4131		Metallophosphoesterase	−0.80	3.06E^−02^	−1.40	1.54E^−05^
SCLAV_4308		Methylmalonyl-CoA epimerase	−0.68	1.77E^−05^	−1.32	1.31E^−15^
SCLAV_4352		Integrin-like protein	1.15	7.63E^−03^	0.42	2.98E^−01^
SCLAV_4355		Hypothetical protein	−0.64	2.99E^−02^	−1.35	< 1E^−06^
SCLAV_4359		Metalloendopeptidase	−2.95	1.92E^−04^	−3.36	< 1E^−06^
SCLAV_4530		Acetiltransferase	2.52	< 1E^−06^	−0.21	6.30E^−01^
SCLAV_4717		Putative hydroxylase	−1.20	6.98E^−03^	−1.71	2.05E^−05^
SCLAV_5249		Membrane protein	1.31	6.40E^−04^	0.20	6.10E^−01^
SCLAV_p0763		Amidohydrolase:Amidohydrolase-like precursor	−0.84	3.63E^−02^	−0.10	7.94E^−01^
SCLAV_p1123		Putative methyltransferase	3.26	< 1E^−06^	3.44	< 1E^−06^
SCLAV_p1142		Rhs family protein	−1.01	< 1E^−06^	−1.77	< 1E^−06^
pSCL2 Plasmid						
SclaA2_010100027605		Helicase	1.02	3.06E^−03^	−0.11	7.59E^−01^
SclaA2_010100027610		Hypothetical protein	0.91	3.35E^−04^	0.14	6.04E^−01^
SclaA2_010100027625		Hypothetical protein	0.91	4.42E^−06^	1.27	< 1E^−06^
SclaA2_010100027920		Transposase	1.00	7.48E^−04^	0.11	7.16E^−01^
SclaA2_010100027930		Hypothetical protein	2.15	< 1E^−06^	1.27	1.21E^−05^
SclaA2_010100027935		Hypothetical protein	1.07	4.80E^−03^	0.86	1.27E^−02^
SclaA2_010100027955		GntR-family regulatory protein	0.85	3.89E^−02^	0.69	5.42E^−02^
SclaA2_010100027975		Hypothetical protein	0.50	2.02E^−02^	0.96	< 1E^−06^
SclaA2_010100027990		Hypothetical protein	0.70	6.76E^−03^	0.11	6.74E^−01^
SclaA2_010100028020		Hypothetical protein	1.64	8.91E^−08^	1.18	5.15E^−05^
SclaA2_010100028015		Phosphatase	0.88	8.96E^−03^	−0.16	6.20E^−01^
SclaA2_010100028325		Hypothetical protein	1.85	< 1E^−06^	0.42	1.64E^−01^
SclaA2_010100028330		Hypothetical protein	2.71	< 1E^−06^	0.98	4.07E^−02^
SclaA2_010100028335		Transferase	2.97	< 1E^−06^	1.82	3.19E^−05^
SclaA2_010100028340		Telomere-associated protein	1.15	6.58E^−03^	0.27	5.12E^−01^
SclaA2_010100028350		Hypothetical protein	0.73	6.58E^−03^	−0.29	2.42E^−01^
SclaA2_010100028360		Hypothetical protein	0.75	3.28E^−02^	0.10	7.66E^−01^
SclaA2_010100028340		Telomere-associated protein	0.79	1.61E^−02^	0.02	9.56E^−01^
pSCL1 Plasmid						
SclaA2_010100027570		Hypothetical protein	1.69	4.80E^−03^	0.24	6.85E^−01^
SclaA2_010100027560		Hypothetical protein	1.95	4.33E^−13^	1.07	1.82E^−05^
SclaA2_010100027550		Hypothetical protein	1.11	1.81E^−02^	−0.22	6.21E^−01^
SclaA2_010100027545		Hypothetical protein	1.11	6.58E^−03^	−0.51	1.73E^−01^

ABC, ATP-binding cassette transporters; ATP, adenosine triphosphate; BLIP, β-lactamase-inhibitory protein; GTP, guanosine triphosphate; NADH, nicotinamide adenine dinucleotide.

These genes belong to the cephamycin C gene cluster, CA gene cluster, clavams, holomycin, cellular differentiation, carbon, nitrogen, amino acids or phosphate metabolism and energy production.

#### CA biosynthesis genes

Transcriptomic studies showed that expression of the four genes of the *ceaS2* to *cas2* operon, encoding enzymes for the early steps of CA biosynthesis, dropped in the mutant to an average M_c_ value of −6.34 in the exponential phase. Expression of *claR*, encoding the regulatory protein ClaR, is under CcaR control (Pérez-Redondo *et al*., 1998; Santamarta *et al*., 2011). This gene was underexpressed in the *ccaR*-deleted mutant (M_c_ −4.61) and concomitantly transcription of the genes for the late steps of the pathway *car*, *gcaS* as well as *cyp*, *orf12*, *orf13*, *orf14*, *oppA2*, *oppA1* and *orf16*, of unknown function, dropped an average M_c_ value of −4.23, being *oat2*, *orf13* and *car* above this value.

Expression of *orf18* and *orf19*, which encode penicillin-binding proteins, *orf20* for a cytochrome P450 and *pcbR*, tentatively involved in β-lactam resistance, was lower in the mutant than in the wild-type strain. All these genes were expressed at higher M_c_ values (average M_c_ −0.88) than the biosynthetic genes in the *ccaR*-mutant.

An interesting finding was made in relation to genes encoding β-lactamase proteins. The *blp* gene, encoding a putative β-lactamase inhibitory protein, which is located downstream of *ccaR*, was clearly underexpressed in the mutant (M_c_ −6.2 and −4.8 in the exponential and stationary phases), which might be due to the absence of the *ccaR* promoter in the *ccaR*-mutant. The second gene, *blip*, encoding a well-characterized β-lactamase inhibitory protein (Doran *et al*., 1990), was also underexpressed in the mutant. This gene, located outside the CA gene cluster, presented M_c_ values of −2.54 and −2.19 in the exponential and stationary phases, respectively.

A gene external to the CA cluster, *adpA*, encoding a regulatory protein involved in antibiotic biosynthesis (López-García *et al*., 2010) was also slightly downregulated.

#### Genes for CA precursors

Clavulanic acid biosynthesis occurs through condensation of arginine and glyceraldehyde-3-phosphate by the carboxyethylargininesynthase, the first enzyme of the CA pathway. *Streptomyces clavuligerus* and *S. coelicolor* probes of genes for arginine biosynthesis and glycerol utilization were present in the microarrays.

The arginine biosynthesis genes were downregulated in the *ccaR*-deleted mutant. Six genes for arginine biosynthesis, located in three separated locations in the *S. clavuligerus* genome, showed average M_c_ values of −2.01 and −2.85 in the exponential and stationary phases respectively. These genes are controlled by the *argR*-encoded regulator ArgR, whose expression decreased to −1.45 and −2.34, at both sampling times.

The co-transcribed genes for glycerol utilization, *glpF1-glpK1-glpD1*, were weakly but significantly affected by the lack of the CcaR regulator, and the same occurred to the *gylR* gene, encoding the glycerol utilization regulatory protein (Baños *et al*., 2009).

However, a positive effect of CcaR on the second cluster for glycerol utilization might occur, because *glpF2* and *glpK2* were underexpressed in the *ccaR*-deleted strain. The most affected gene in this group was *gap2*, encoding glyceraldehyde-3-phosphate dehydrogenase (M_c_ −2.61 in the exponential growth phase).

#### Cephamycin C biosynthesis genes

In relation to cephamycin C biosynthesis, the transcriptomic data fitted well with those previously obtained by quantative reverse transcription polymerase chain reaction (Santamarta *et al*., 2011). The M_c_ values for *lat*, *cmcI*, *cmcJ*, *cefD* and *cefF* genes, either starting transcriptional units or close to the first gene of the operon, showed values ranging from −6.15 to −7.48 in the exponential phase, close to the −7.12 value of *ccaR* (which is deleted in the mutant), indicating a complete lack of transcription of these units. All other genes related to cephamycin biosynthesis showed M_c_ values between −3.2 and −7.12 in the exponential phase and their expression dropped in the *ccaR*-mutant by an average of 18% in the stationary phase.

The *pbp74* and *bla* genes were barely affected in the exponential phase but were less expressed in the mutant in the stationary phase.

#### Genes for other antibiotics produced by *S. clavuligerus*

All the holomycin biosynthesis genes tested were overexpressed in the *ccaR*-deleted mutant, with an average M_c_ value of 5.57 in the exponential phase, while the regulatory gene *hlmM* had a M_c_ value of 2.01. These results coincide with the formation of holomycin by the *S. clavuligerus ccaR::aph* disrupted mutant detected by Fuente and colleagues (2002). A downregulation was observed in the clavams and CA paralogous clusters, especially in the stationary phase. Production of clavams is relatively variable and medium-dependent. However, *cas1* encoding the second clavaminate synthase isoenzyme (M_c_ −3.32 and −4.29) and *cvm8*, encoding a serine hydroxymethyltransferase (M_c_ −2.59 and −3.18) were strongly downregulated in the *ccaR-*deleted mutant.

### Nitrogen and phosphate metabolism

The lack of CcaR and, therefore, of CA and cephamycin C formation, affected nitrogen metabolism. A strong downregulation was observed in the exponential phase in the expression of the ammonium transporter-encoding gene *amtB* (M_c_ −4.1) and the downstream gene, *glnB* (M_c_ −4.39), which encodes the uridilyltransferase regulatory protein PII. This effect was stronger in the stationary phase (M_c_ −5.04 and −5.09, for *amtB* and *glnB* respectively). Also genes for glutamine synthetases (*glnA3*, *glnA2*, *glnA1*) were underexpressed in the *ccaR*-deleted mutant (M_c_ −4.67, −3.56 and −1.77) in the exponential phase. A weaker, but clear downregulation also occurred in *glu*D1, encoding a glutamate permease. The homologous probes of *S. coelicolor* (also present in the arrays) for glutamine synthetase (*glnA*, *glnII*, SCO1613) as well of genes for urease (*ureAB*) and an allantoinase (SCO6247) gave a lower signal when hybridized with *S. clavuligerus* Δ*ccaR::tsr* messenger ribonucleic acid (mRNA) in relation to *S. clavuligerus* ATCC 27064 mRNA. This might indicate that cephamycin C and CA production requires a strong demand of nitrogen-derived precursors and, therefore, nitrogen metabolism slows down when antibiotic production is blocked.

Some genes involved in phosphate transport (*pstA*, *pstC and pstS*) showed a weak but clear underexpression in the *ccaR*-mutant, especially in the stationary phase sampling point.

### Miscellaneous genes

Several genes involved in energy production or lipid metabolism were affected in the *ccaR*-deleted strain. This was the case for the genes encoding putative acetyl-CoA synthetases and acetyl-CoA carboxylases (SCLAV_4986, SCLAV_3405, SCLAV_3406) and the same occurred with the acetyltransferase-and methyltransferase-encoding genes (SCLAV_4530 and SCLAV_p1123). A set of genes whose function is unknown (SclaA2_010100027930, SclaA2_010100028330 and SclaA2_010100028335) all located in plasmid pSCL2, showed a clear overexpression in the mutant in the exponential phase of growth.

#### Effect on regulatory genes

The transcription of 13 genes encoding different types of regulators was affected in the mutant, including *adpA* (see previous discussion). A very strong downregulation was observed for the *whiB*-family transcriptional regulator (M_c_ 2.62) and for the gene encoding an AmphR1-like regulator (SCLAV_5278) with an M_c_ value of 2.0 in the exponential phase.

### Expression of *S. clavuligerus* genes as detected by transcriptomic analysis of orthologous *S. coelicolor* genes

The whole *S. coelicolor* genome was represented in the microarrays. Therefore, we were able to compare expression of miscellaneous genes by heterologous hybridization of *S. coelicolor* probes present in the microarray with mRNA from *S. clavuligerus*. Heterologous transcriptomic studies confirmed the lower expression of the *glnA*, *glnII*, SCLAV_0834, *ureAB* and *gap2* genes in the Δ*ccaR* mutant. Other blocks of underexpressed genes were those encoding succinate dehydrogenases (SCLAV_3969 and SCLAV_3970) and the *nuo* genes (*nuoEFJKLMN*) for different subunits of the nicotinamide adenine dinucleotide (NADH) dehydrogenase. Genes for membrane proteins or for hypothetical proteins were also found to be underexpressed; but some were overexpressed, including the orthologous aminotransferase encoded by SCLAV_5663 (M_c_ 2.63) and the methyltransferase encoded by SCLAV_5654 (M_c_ 2.56) (Supporting Information Table S1).

### Validation studies

#### Quantitative reverse transcription polymerase chain reaction (qRT-PCR)

The validation of many genes differentially expressed in this transcriptomic analysis has already been carried out using proteomics or qRT-PCR (Santamarta *et al*., 2011). Such is the case for all the downregulated genes of the CA pathway and for the *lat*, *cmcI*, *cefD* or *cmcT* genes in the cephamycin C gene cluster.

**Figure 2:**
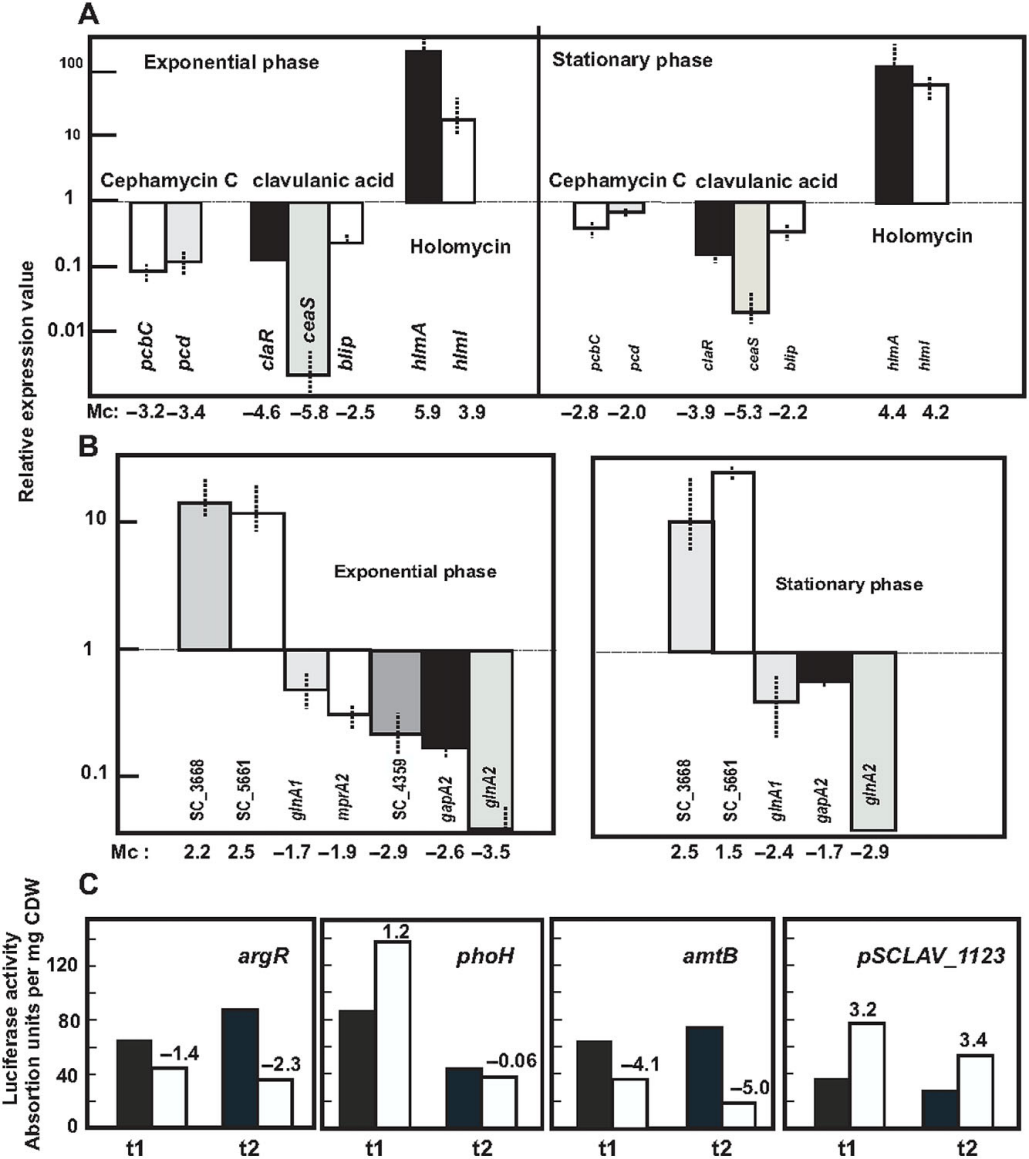
Validation of the microarray results by qRT-PCR. Quantitative RT-PCR of genes indicated below using the oligonucleotides shown in Supporting Information Table S2. The relative values are referred to 1, assigned relative value for the expression of each gene in *S. clavuligerus* ATCC 27064. Error bars were calculated by measuring the standard deviation among biological replicates of each sample. A. Antibiotic biosynthesis genes. B. Miscellaneous genes indicated below. C. Validation of the microarray data using the luciferase coupled method. *Streptomyces clavuligerus* ATCC 27064 (black bars); *S*. *clavuligerus* Δ*ccaR::tsr* (white bars). The numbers indicate for each gene the relative M_c_ values of *S*. *clavuligerus* Δ*ccaR::tsr* for each gene.

Other differentially regulated genes present in Table 1 and Supporting Information Table S1 were validated by qRT-PCR performed on reverse-transcribed RNA samples. A total of 15 genes (*pcbC*, *pcd*, *claR*, *ceaS2*, *blip*, *hlmI*, *hlmA*, *gapA2*, *glnA1*, *glnA2*, SCLAV_4359, SCLAV_3668, *mprA2*, SCLAV_5661 and, as control, *hrdB*) were chosen for analysis (Fig. 2). The Pearson's correlation coefficient (R^2^ = 0.9243) between M_c_ values and relative expression values of qRT-PCR indicates a good validation of the results.

#### Luciferase activity

Promoters of the *argR*, *phoH*, *amtB* and SCLAV_p1123 genes were coupled to the *luxAB* genes and the constructions were introduced in *S. clavuligerus* ATCC 27064 and *S. clavuligerus* Δ*ccaR::tsr.* Luciferase activity was lower in *S. clavuligerus* Δ*ccaR::tsr* when the *luxAB* genes were expressed from the *argR* and *phoH* promoters (Fig. 2C) and higher when expression was carried out from the *amtB* and SCLAV_p1123 promoters in agreement with the results obtained in the transcriptomic experiments. The correlation index between the data obtained, measuring luciferase activity and the M_c_ values of the respective genes, was 0.796. Luciferase activity differed slightly from the expected data of the microarrays when the enzyme was formed in the stationary phase using the *phoH* promoter.

## Discussion

A transcriptomic study reveals the complete gene expression in a micro-organism, allowing the comparison of overproducing strains and wild-type strains (Medema *et al*., 2010). However, while this method is convenient for understanding the metabolic flow in industrial strains, these strains have been obtained through heavy mutagenesis and selection programs; therefore, the final results observed might not be targeted at a particular mutation but derive from indirect effects of randomly mutated genes.

In this study, we compared the wild-type strain *S. clavuligerus* ATCC 27064 and a *ccaR*-deleted mutant obtained by molecular methods (Alexander and Jensen, 1998). As expected, the CA and cephamycin C biosynthesis genes are strongly downregulated in the mutant in agreement with the activator effect found previously for CcaR (Pérez-Llarena *et al*., 1997; Santamarta *et al*., 2011). These differences are very marked (a 37-fold decrease in the exponential phase) for all the CA biosynthesis genes, or the cephamycin biosynthesis genes (559-fold) but lower (average 1.6 fold) for genes located at the ends of the clusters (*orf18*, *orf19*, *orf20*, *pcbR*, *pbpA*), which are supposed to be involved in β-lactam antibiotic resistance. Interestingly, two genes encoding β-lactamase inhibitory proteins, *blip* and *blp*, are downregulated in the mutant. This is the first description of a relationship between CcaR and *blip* expression, a gene located outside the CA cluster. According to the transcriptomic data, *blip* is expressed as an independent gene. A putative heptameric sequence for CcaR binding is present 104 bp upstream of *blip*, suggesting a direct effect of CcaR on the expression of this gene; however, electrophoretic mobility shift assay analysis has to be performed to confirm this hypothesis. The function of the *blp*-encoded protein has not been clearly elucidated (Thai *et al*., 2001), but this gene is strongly downregulated in the *ccaR*-mutant, perhaps due to a co-transcription with the *ccaR* gene located upstream.

Medema and colleagues (2010) found several uncharacterized gene clusters, including one for secondary metabolism, that were overexpressed in the high-producing strain. The opposite is found in *S. clavuligerus* Δ*ccaR*::*tsr*, in which all the genes for holomycin biosynthesis (*hlmA* to *hlmM*) are overexpressed (average values of 32-fold) confirming that the *ccaR*-knockout mutant produces more holomycin than the wild-type strain (Fuente *et al*., 2002; Robles-Reglero *et al*., 2013). These results agree with those of Chen and colleagues (2009) who reported an overexpression of the filipin biosynthesis genes in an *ave1*-disrupted mutant unable to produce avermectin and suggest that precursor and energy flow might be directed to the production of a different secondary metabolite once the most abundant metabolite pathway has been disrupted.

Expression of the clavams biosynthesis cluster or the ‘paralogous’ CA cluster, including the *cvm7 gene*, encoding a LAL regulator, was weakly downregulated except for the *cas1* and *orf8* genes, encoding the clavaminate synthetase1 isoenzyme and a serine hydroxymethyltransferase, which were strongly downregulated. This result contrasts with that of Medema and colleagues (2010) who described a significant overexpression of all these genes in the CA overproducing mutant that overexpresses *ccaR*.

The effects of CcaR absence on the transcription of *S. clavuligerus* primary metabolism genes are validated by the parallel transcription results for *S. coelicolor* genes present in the microarray.

Clavulanic acid is formed from arginine and a C3 compound derived from glycerol. All genes for arginine biosynthesis are strongly downregulated in the mutant suggesting that cells detect the lack of arginine requirement for CA biosynthesis in the *ccaR*-deleted mutant and shut down the arginine pathway. Genes for glycerol utilization (*glpF2, glpK2*) are expressed in the CA non-producing mutants in the order of 0.4-fold in relation to the control strain, which is a small reduction if compared with the two-fold expression increase observed for these genes by Medema and colleagues (2010) in the high CA-producing strain; however, our result in the *gap2* gene, for glyceraldehyde-3-phosphate dehydrogenase 2, indicates that this gene is downregulated (0.16-fold), confirming what was observed by Medema and colleagues (2010) in the high CA-producing strain and the relevance of *gap2*-disruption in increasing the glyceraldehyde-3-phoshate required for CA production (Li and Townsend, 2006).

In summary, our results confirm most of those previously obtained for a CcaR overproducing strain. CcaR binds heptameric sequences in many CA and cephamycin C genes. However, no clear heptameric sequences have been found in nitrogen metabolism genes, genes controlling energy flow or genes for the antibiotic precursors suggesting that the lack of CA and cephamycin C production directly affects the flow of these pathways.

## Experimental procedures

### Culture conditions

*Streptomyces clavuligerus* ATCC 27064 and the mutants *S. clavuligerus ΔccaR::tsr* (Alexander and Jensen, 1998) and *S. clavuligerus ccaR::aph* (Pérez-Llarena *et al*., 1997) were used in this work. Strain *S. clavuligerus ΔccaR::tsr* was chosen for the transcriptomic experiments because the *ccaR* was deleted. To determine the more homogeneous and repetitive conditions for RNA sampling, the wild-type strain and *S. clavuligerus* Δ*ccaR::tsr* were grown in SA medium and DNA, cell dry weight and antibiotics production were analyzed. Trypticase soy broth medium (100 ml) was inoculated with 1 ml of frozen mycelia, and the culture was grown to an OD_600nm_ of 6.5. This culture was used to inoculate (5% v/v) 500 ml baffled flasks containing 100 ml of semidefined SA medium (Aidoo *et al*., 1994). The cultures were maintained at 28°C with 220 r.p.m. shaking. Exponential phase sampling was done when the cultures reached a DNA content of 75 and 80 μg ml^−1^ in the wild type and the mutant (24 and 32 h respectively) and 160 and 170 μg DNA ml^−1^ in the wild type and the mutant for the early stationary phase (40 and 50 h respectively) (Fig. 1).

### RNA isolation and purification

Samples (2 ml) from *S. clavuligerus* ATCC 27064 and *S. clavuligerus ΔccaR::tsr* in the exponential and stationary growth phase in SA medium were stabilized with two volumes of RNA Protect Bacteria Reagent (Qiagen) for 5 min, then 1% β-mercaptoethanol was added. The samples were treated as indicated by Álvarez-Álvarez and colleagues (2013).

### Labelling and microarray hybridizations

*Streptomyces clavuligerus* microarrays were obtained from Agilent Technologies (Santa Clara, CA, USA), in the Agilent 8 × 15K format. They include *S. clavuligerus* quadruple probes for about 800 genes and intergenic regions of some clusters involved in secondary metabolism, and also duplicated probes for 7728 chromosomal genes (out of 7825) of the *S. coelicolor* genome. Four biological replicates were made for each condition (two strains and two growth times). Labelling reactions were performed according to the recommendations described by BioPrime Array CGH Genomic Labelling Systems (Life Technologies, Carlsbad, CA, USA). Total RNA was labelled with Cy3-dCTP (Amersham, Freiburg, Germany) using random primers and Superscript II reverse transcriptase (Invitrogen, Carlsbad, CA, USA). Genomic deoxyribonucleic acid (gDNA) was labelled with Cy5-dCTP (Amersham) from random primers extended with the Klenow fragment of DNA polymerase (Roche, Basel, Switzerland). The final products were purified with MinElute columns (Qiagen, Venlo, the Netherlands) and labelling efficiencies were quantified spectrophotometrically. Cy3-cDNA (300 ng) and Cy5-labelled gDNA (10 pmol) were mixed, vacuum dried, resuspended in 32 μL of hybridization solution (Agilent) and applied on the microarray surface. Hybridizations were carried out at 55°C and extended to 60 h to improve the quality of the results (Sartor *et al*., 2004). Washing, scanning with an Agilent DNA Microarray Scanner G2565BA and image quantification were carried out as indicated previously (Rodríguez-García *et al*., 2007).

### Identification of differentially transcribed genes and transcription profile classification

Microarray data were normalized and analyzed with the Bioconductor package limma (Smyth and Speed, 2003; Smyth, 2004). Weighted median was applied within arrays. Weights were assigned as follows: 1, probes corresponding to *S. coelicolor* genes showing a raw Cy3 intensity value higher than 2000; 0.25, probes corresponding to *S. clavuligerus* genes; and 0, the remaining probes. The normalized log_2_ of Cy3/Cy5 intensities is referred to as the M_g_ value, which is proportional to the abundance of transcripts for a particular gene (Mehra *et al*., 2006). The procedure by Smyth and colleagues (2005) to include the information from within-array replicates was applied to the set of quadruple probes. The M_g_ transcription values of the four experimental conditions were compared using two contrasts, mutant *versus* wild type, corresponding to the two studied growth phases (exponential and stationary). For each gene, the M_c_ value is the binary log of the differential transcription between the mutant and the wild strain. A positive M_c_ value indicates upregulation, and a negative one, downregulation. False discovery rate (FDR) correction for multiple testing was applied. A result was considered as statistically significant if the FDR-corrected *P*-value < 0.05. The microarray data have been deposited in National Center for Biotechnology Information-Gene Expression Omnibus under accession number GSE51435.

### qRT-PCR

The oligonucleotide primers used in this work are shown in Table S2 (Supporting Information). All PCR reactions were performed using total DNA of *S. clavuligerus* strains as a template in a T-gradient (Biometra, Goettingen, Germany) thermocycler. The PCR reaction (30 μl) was performed as described by Kieser and colleagues (2000), and contained 300 ng DNA template, 0.5 mM each oligonucleotide, 28 mM each dGTP and dCTP, 12 mM each dATP and dTTP, 1 mM MgCl_2_, dimethylsulfoxide 5%, and 0.8 U Taq DNA polymerase. The amplification programme was as follows: after a step of 95°C for 30 s, the annealing temperature was reduced in a touchdown of 1°C from 60°C to 55°C in one cycle, with an annealing time of 30 s; an annealing temperature of 55°C was used in the next 25 cycles with an extension step of 1 min at 72°C. Quantification and purity analysis of all PCR products was determined using a NanoDrop ND-1000 Spectrophotometer (Thermo Scientific, Waltham, MA, USA).

Gene expression analysis by qRT-PCR was performed as previously described (López-García *et al*., 2010). *Streptomyces clavuligerus* RNA was obtained in the same way as that used for microarray experiments. RNA samples were prepared using RNeasy mini-spin columns. The samples were treated with DNase I (Qiagen) and Turbo DNase (Ambion, Carlsbad, CA, USA) to eliminate DNA. Negative controls on qRT-PCR amplification (to confirm the absence of contaminating DNA) were carried out with each set of primers. The efficiency of the primers used was measured by serial dilution of genomic DNA as template. Relative quantification of gene expression was performed by the ΔΔC_t_ method (Livak and Schmittgen, 2001), using the constitutive housekeeping gene *hrdB* as reference (Buttner *et al*., 1990).

### Luciferase assay

For luciferase reporter analysis, promoter regions were amplified with primers containing NdeI and BamHI restriction sites (Supporting Information Table S2) to clone the promoters in the ATG codon of the *luxA* gene in pLUXAR-neo. Cultures of *S. clavuligerus* exconjugants harbouring the promoter–probe constructs were carried out in SA medium. Sample treatment and the luciferase assays were done as described by Pérez-Redondo and colleagues (2012). At least two different cultures from the same strain were analyzed for luminescence production and measured in triplicate.

## Supporting information

Table S1 Expression of *S. clavuligerus* genes as detected by hybridization in microarrays with *S. coelicolor* probes

Table S2 Oligonucleotides used in this work
