# Supplementary material for: Transcriptomic analysis of Streptomyces clavuligerus ΔccaR::tsr: effects of the cephamycin C-clavulanic acid cluster regulator CcaR on global regulation
Source: Microb Biotechnol. 2014 Jan 22;7(3):221–31. doi: 10.1111/1751-7915.12109 (PMC3992018; doi:10.1111/1751-7915.12109)
Supplement: Table S1 — Expression of S. clavuligerus genes as detected by hybridization in microarrays with S. coelicolor probes [file mbt20007-0221-sd1.doc]

**Transcriptomic analysis of *Streptomyces clavuligerus* Δ*ccaR::tsr*: effects of CcaR in global regulation.**

Álvarez-Álvarez, R.^1,2^, Rodríguez-García, A.^1,2^, Santamarta, I.^2^, Pérez-Redondo, R.^2^, Prieto-Domínguez, A.^1^ , Martínez-Burgo, Y.^1^, and Liras, P.^1,2^

^1^ Área de Microbiología, Departamento de Biología Molecular. Facultad de CC. Biológicas y Ambientales. Universidad de León, 24071 León, Spain.

^2^ Instituto de Biotecnología de Léon (INBIOTEC). Parque Científico de León, Avda. Real nº1, 24006 León, Spain.

**Table S1. Expression of *S. clavuligerus* genes as detected by hybridization in microarrays with *S.coelicolor* probes**

|  |  | | |  | |  | |  | | **EXPONENTIAL PHASE** | | | **STATIONARY PHASE** | |
| --- | --- | --- | --- | --- | --- | --- | --- | --- | --- | --- | --- | --- | --- | --- |
|  | **CODE** | | **ORTHOLOG** | | **GENE** | | **PRODUCT** | | | **Mc** | **FDR** | **Mc** | | **FDR** |
|  | ***Energy*** | |  | |  | |  | | |  |  |  | |  |
|  | SCO5106 | | SCLAV_3969 | | ***dhsB2**** | | Putative succinate dehydrogenase iron-sulfur subunit | | | **-1,60** | 5,81E-03 | **-1,34** | | 3,10E-03 |
|  | SCO5107 | | SCLAV_3970 | | ***dhsA2**** | | Putative succinate dehydrogenase flavoprotein subunit | | | **-1,19** | 3,60E-02 | **0,00** | | 9,93E-01 |
|  | SCO4566 | | SCLAV_3568 | | ***nuoE*** | | NuoE, NADH dehydrogenase subunit E | | | **-1,10** | 3,99E-02 | **-1,06** | | 4,50E-03 |
|  | SCO4567 | | SCLAV_3569 | | ***nuoF*** | | NuoF, NADH dehydrogenase subunit F | | | **-1,14** | 4,13E-02 | **-0,32** | | 3,43E-01 |
|  | SCO4571 | | SCLAV_3571 | | ***nuoJ*** | | NuoJ, NADH dehydrogenase subunit J | | | **-1,10** | 2,87E-02 | **-0,51** | | 1,07E-01 |
|  | SCO4572 | | SCLAV_3574 | | ***nuoK*** | | NuoK, NADH dehydrogenase subunit K | | | **-1,27** | 8,21E-03 | **-0,17** | | 6,06E-01 |
|  | SCO4573 | | SCLAV_3575 | | ***nuoL*** | | NuoL, NADH dehydrogenase subunit L | | | **-1,09** | 4,93E-02 | **-0,15** | | 6,59E-01 |
|  | SCO4574 | | SCLAV_3576 | | ***nuoM*** | | NuoM, NADH dehydrogenase subunit M | | | **-1,14** | 1,28E-02 | **-0,55** | | 7,32E-02 |
|  | SCO4575 | | SCLAV_3577 | | ***nuoN*** | | NuoN, NADH dehydrogenase subunit N | | | **-1,14** | 6,13E-03 | **-0,10** | | 7,35E-01 |
|  | ***Carbon Metabolism*** | | | | | | | |  | | |  | | |
|  | SCO4380 | | SCLAV_3405 | |  | | Putative acetyl/propionyl CoA carboxylase, beta subunit | | | **-1,54** | 2,36E-04 | **-2,16** | | <1E-06 |
|  | SCO7040 | | SCLAV_5509 | | ***gap2**** | | Glyceraldehyde-3-phosphate dehydrogenase | | | **-2,12** | <1E-06 | **-1,44** | | 2,22E-05 |
|  | SCO6497 | | SCLAV_5490 | | ***tktA2*** | | Transketolase A | | | **2,58** | 7,76E-04 | **3,18** | | <1E-06 |
|  | ***Nitrgen Metabolism*** | | | | | | | |  | | |  | | |
|  | SCO1613 | | SCLAV_0834 | |  | | Putative glutamine synthetase | | | **-1,56** | 1,28E-02 | **-1,61** | | 9,83E-04 |
|  | SCO2198 | | SCLAV_1416 | | ***glnA*** | | Glutamine synthetase I | | | **-3,67** | 1,52E-05 | **-3,18** | | 3,02E-05 |
|  | SCO2210 | | SCLAV_1431 | | ***glnII*** | | Glutamine synthetase | | | **-5,01** | 4,01E-04 | **-5,11** | | 4,66E-05 |
|  | SCO5525 | | SCLAV_4473 | | ***ureAB*** | | Fusion of urease beta and gamma subunits | | | **-1,47** | 3,47E-05 | **-1,12** | | 2,02E-04 |
|  | SCO6247 | | SCLAV_5009 | |  | | Putative allantoinase | | | **2,59** | 6,27E-05 | **1,85** | | 6,12E-04 |
|  | ***Transport Protein*** | | | | | | | |  | | |  | | |
|  | SCO0742 | | SCLAV_1387 | |  | | ABC transporter,putative ABC transporter | | | **-1,06** | 2,71E-02 | **-0,02** | | 9,60E-01 |
|  | SCO1900 | | SCLAV_1521 | |  | | Putative integral membrane sugar transport protein | | | **-1,20** | 2,91E-03 | **-0,90** | | 4,84E-03 |
|  | SCO2463 | | SCLAV_1669 | |  | | Putative ABC transporter | | | **1,21** | 1,99E-02 | **1,32** | | 7,85E-04 |
|  | SCO2631 | | SCLAV_1802 | |  | | Putative amino acid permease | | | **-1,83** | 1,89E-04 | **-2,08** | | <1E-06 |
|  | | SCO4148 | SCLAV_1662 | |  | | Putative ABC transport system ATP-binding protein | | | **2,00** | 8,19E-03 | **2,20** | | 2,89E-04 |
|  | | SCO4964 | SCLAV_3858 | |  | | Putative integral membrane transport protein | | | **1,68** | 6,98E-04 | **1,42** | | 6,06E-04 |
|  | | SCO5400 | SCLAV_4310 | |  | | Putative transport system kinase | | | **1,30** | 7,39E-04 | **0,59** | | 3,80E-02 |
|  | | SCO6054 | SCLAV_p0702 | |  | | Putative transmembrane transport protein | | | **-1,11** | 4,57E-03 | **-1,09** | | 5,69E-04 |
|  | | SCO6512 | SCLAV_3668 | |  | | ABC transporter ATP-binding protein | | | **2,29** | <1E-06 | **2,57** | | <1E-06 |
|  | | ***Membrane Protein*** | | | | | | | |  |  |  | |  |
|  | | SCO0384 | SCLAV_5647 | |  | | Putative membrane protein | | | **1,25** | 3,18E-02 | **1,42** | | 8,15E-04 |
|  | | SCO0390 | SCLAV_5652 | |  | | Putative membrane protein | | | **2,40** | 1,52E-05 | **0,68** | | 8,66E-02 |
|  | | SCO0399 | SCLAV_5661 | |  | | Putative membrane protein | | | **2,52** | <1E-06 | **1,53** | | 9,61E-04 |
|  | | SCO5108 | SCLAV_3971 | |  | | putative integral membrane protein | | | **-2,07** | 2,24E-03 | **-0,59** | | 2,08E-01 |
|  | | SCO5123 | SCLAV_3987 | |  | | Putative small membrane protein | | | **-1,16** | 1,74E-02 | **-1,82** | | 1,13E-05 |
|  | | SCO6427 | SCLAV_1492 | |  | | Putative integral membrane transport protein | | | **1,80** | 7,39E-04 | **1,83** | | 9,07E-05 |
|  | | ***Secreted Protein*** | | | | | | | |  |  |  | |  |
|  | | SCO0677 | SCLAV_4996 | | ***csn*** | | Secreted chitosanase | | | **1,66** | 7,58E-03 | **1,64** | | 8,55E-04 |
|  | | SCO7432 | SCLAV_4112 | | ***mprA2*** | | Secreted extracellular small neutral protease. | | | **-1,98** | 1,16E-02 | **-2,32** | | 2,06E-04 |
|  | | SCO7532 | SCLAV_5381 | |  | | Putative secreted protein. | | | **1,95** | 6,12E-04 | **1,26** | | 5,93E-03 |
|  | | ***Stress*** |  | |  | |  | | |  |  |  | |  |
|  | | SCO4325 | SCLAV_3305 | | ***cspB*** | | Cold shock protein B | | | **-1,14** | 4,15E-03 | **-1,49** | | 2,21E-05 |
|  | | SCO5032 | SCLAV_3935 | | ***ahpC*** | | Alkyl hydroperoxide reductase | | | **1,87** | 2,97E-03 | **1,86** | | 3,46E-04 |
|  | | ***Translation*** |  | |  | |  | | |  |  |  | |  |
|  | | SCO3961 | SCLAV_2841 | | ***serS*** | | Seryl-tRNA synthase | | | **1,24** | 2,63E-02 | **0,20** | | 5,61E-01 |
|  | | SCO5708 | SCLAV_4604 | |  | | Putative ribosome-binding factor | | | **1,02** | 4,93E-02 | **-0,16** | | 6,13E-01 |
|  | | SCO5709 | SCLAV_4605 | |  | | Probable tRNA pseudouridine synthase | | | **1,28** | 9,16E-03 | **-0,22** | | 5,10E-01 |
|  | | SCO5699 | SCLAV_4595 | | ***proS*** | | Prolyl tRNA synthetase | | | **1,14** | 4,93E-02 | **0,08** | | 8,35E-01 |
|  | | ***Transcriptional Regulator*** | | | | | | | |  |  |  | |  |
|  | | SCO1200 | SCLAV_2714 | |  | | Putative regulatory protein | | | **1,18** | 1,85E-03 | **0,71** | | 1,51E-02 |
|  | | SCO2508 | SCLAV_1702 | | ***zur*** | | Putative metal uptake regulation protein | | | **-1,41** | 5,73E-05 | **-0,99** | | 6,89E-04 |
|  | | SCO2792 | SCLAV_1957 | | ***adpA*** | | AraC-family transcriptional regulator (ArpA target) | | | **-1,14** | 4,67E-02 | **-1,25** | | 1,77E-03 |
|  | SCO5351 | | SCLAV_4247 | |  | | Putative regulatory protein | | | **1,06** | 4,93E-02 | **0,48** | | 1,39E-01 |
|  | SCO6770 | | SCLAV_5241 | |  | | Putative DNA-binding protein | | | **-1,14** | 1,19E-02 | **-1,38** | | 1,54E-04 |
|  | SCO7533 | | SCLAV_5379 | |  | | Two-component system response regulator | | | **1,20** | 1,79E-02 | **-0,34** | | 2,99E-01 |
|  | ***Unknown Function*** | | | | | | | | |  |  |  | |  |
|  | SCO0382 | | SCLAV_5645 | |  | | UDP-glucose/GDP-mannose family dehydrogenase | | | **3,36** | <1E-06 | **1,80** | | 1,93E-04 |
|  | SCO1612 | | SCLAV_0833 | |  | | Putative aldehyde dehydrogenase | | | **-1,67** | 6,46E-04 | **-2,64** | | <1E-06 |
|  | SCO1611 | | SCLAV_0832 | |  | | Putative dehydrogenase | | | **-1,90** | 1,85E-03 | **-2,12** | | 5,98E-05 |
|  | SCO1761 | | SCLAV_0974 | |  | | Putative cyclohexadienyl dehydrogenase | | | **1,29** | 3,63E-02 | **1,86** | | 7,39E-05 |
|  | SCO0392 | | SCLAV_5654 | |  | | Putative methyltransferase | | | **2,56** | 1,49E-07 | **1,65** | | 5,03E-05 |
|  | SCO0393 | | SCLAV_5655 | |  | | Putative transferase | | | **2,28** | 4,20E-06 | **0,89** | | 1,81E-02 |
|  | SCO0398 | | SCLAV_5660 | |  | | Putative glycosyl transferase | | | **2,14** | 4,46E-04 | **2,94** | | 1,11E-06 |
|  | SCO0401 | | SCLAV_5663 | |  | | Putative aminotransferase | | | **2,63** | 2,36E-08 | **1,63** | | 1,81E-05 |
|  | SCO1250 | | SCLAV_0535 | |  | | Putative acetyltransferase | | | **1,71** | 9,55E-05 | **0,26** | | 3,93E-01 |
|  | SCO7447 | | SCLAV_p0264 | |  | | Putative acetyltranferase. | | | **1,61** | 6,12E-04 | **1,17** | | 2,26E-03 |
|  | SCO0383 | | SCLAV_5646 | |  | | Hypothetical protein SCF62.09 | | | **3,18** | 1,71E-07 | **1,32** | | 5,28E-03 |
|  | SCO0387 | | SCLAV_5650 | |  | | Hutative bi-domain oxidoreductase | | | **1,96** | 6,81E-04 | **1,05** | | 1,89E-02 |
|  | SCO0388 | | SCLAV_5651 | |  | | Hypothetical protein SCF62.14 | | | **3,08** | 1,70E-07 | **1,78** | | 1,98E-04 |
|  | SCO0395 | | SCLAV_5657 | |  | | Putative epimerase/dehydratase | | | **2,38** | 3,88E-05 | **0,82** | | 5,40E-02 |
|  | SCO1695 | | SCLAV_0924 | |  | | Hypothetical protein | | | **-1,79** | 3,92E-03 | **-1,81** | | 3,56E-04 |
|  | SCO1726 | | SCLAV_0935 | |  | | Putative ATPase | | | **1,07** | 3,78E-02 | **0,86** | | 1,48E-02 |
|  | SCO1898 | | SCLAV_1523 | |  | | Putative substrate binding protein | | | **-1,41** | 8,62E-04 | **-1,06** | | 2,23E-03 |
|  | SCO2397 | | SCLAV_1635 | |  | | Putative oxidoreductase | | | **1,12** | 3,66E-02 | **1,36** | | 4,90E-04 |
|  | SCO2460 | | SCLAV_2637 | |  | | Conserved hypothetical protein SCC24.31c | | | **1,75** | 2,75E-03 | **1,65** | | 5,53E-04 |
|  | SCO2547 | | SCLAV_1722 | |  | | Putative hydrolase | | | **-1,03** | 1,09E-02 | **-0,51** | | 6,33E-02 |
|  | SCO2966 | | SCLAV_2075 | | ***smpB*** | | Small protein B homologue | | | **1,15** | 6,21E-03 | **-0,78** | | 1,46E-02 |
|  | SCO2978 | | SCLAV_2084 | |  | | Putative secreted protein | | | **-1,16** | 1,82E-02 | **-2,19** | | 1,11E-06 |
|  | SCO3581 | | SCLAV_2575 | |  | | Conserved hypothetical protein | | | **1,46** | 1,85E-03 | **0,06** | | 8,62E-01 |
|  | SCO3636 | | SCLAV_2638 | |  | | Putative cytochrome P-450 hydroxylase | | | **1,13** | 4,39E-03 | **0,73** | | 1,57E-02 |
|  | | SCO5191 | SCLAV_4055 | |  | | Hypothetical protein 2SC3B6.15 | | | **2,87** | 5,28E-06 | **-0,05** | | 9,21E-01 |
|  | | SCO5285 | SCLAV_4136 | | ***lon*** | | ATP-dependent protease | | | **1,16** | 1,28E-02 | **0,31** | | 3,11E-01 |
|  | | SCO5376 | SCLAV_4288 | | ***chiC*** | | Chitinase C (putative secreted protein) | | | **-1,07** | 7,58E-03 | **-1,77** | | 2,07E-06 |
|  | | SCO5796 | SCLAV_4676 | |  | | Conserved hypothetical protein SC4H2.17 | | | **1,74** | 2,06E-04 | **0,31** | | 3,43E-01 |
