# Supplementary material for: Transcriptomic analysis of Streptomyces clavuligerus ΔccaR::tsr: effects of the cephamycin C-clavulanic acid cluster regulator CcaR on global regulation
Source: Microb Biotechnol. 2014 Jan 22;7(3):221–31. doi: 10.1111/1751-7915.12109 (PMC3992018; doi:10.1111/1751-7915.12109)
Supplement: Table S2 — Oligonucleotides used in this work [file mbt20007-0221-sd2.doc]

**Transcriptomic analysis of *Streptomyces clavuligerus* Δ*ccaR::tsr*: effects of CcaR in global regulation.**

Álvarez-Álvarez, R.^1,2^, Rodríguez-García, A.^1,2^, Santamarta, I.^2^, Pérez-Redondo, R.^2^, Prieto-Domínguez, A.^1^ , Martínez-Burgo, Y.^1^, and Liras, P.^1,2^

^1^ Área de Microbiología, Departamento de Biología Molecular. Facultad de CC. Biológicas y Ambientales. Universidad de León, 24071 León, Spain.

^2^ Instituto de Biotecnología de León (INBIOTEC). Parque Científico de León, Avda. Real nº1, 24006 León, Spain.

**Table S2. Oligonucleotides used in this work**

|  | **Sequence (5´ to 3´)** | **Product size (bp)** | **Annealing temperature (ºC)** |
| --- | --- | --- | --- |
| Q*pcbC*-D | GACCGACCAGGAGAAGCAC |  |  |
| Q*pcbC*-R | CGGGTTGAGGTAACAGAAGG | 127 | 62 |
| Q*pcd*-D | CTGGGCGTGGTCGGTGT |  |  |
| Q*pcd*-R | GCGGTCAGCGGGGTCAG | 128 | 64 |
| Q*blip-*D | GTCGTTCGGGGACAGCA |  |  |
| Q*blip-*R | AACTTGGCGAGGGTGAG | 162 | 64 |
| Q*gap2*-D | ATCCTCAAGACCCACCGTTT |  |  |
| Q*gap2*-R | GTGAACTCCTCCACCGTCAG | 179 | 62 |
| Q*glnA2*-D | GGACGGGAGTAGGAGGAGTT |  |  |
| Q*glnA2*-R | AAACCACGGATGGACGAG | 192 | 64 |
| Q*glnA1*-D | GACGGCTCCAGGGCAAG |  |  |
| Q*glnA1*-R | GACGGTGTTCATCTCGGTGT | 113 | 64 |
| QSclav_4359-D | ACGCCTTCTGGACCGACA |  |  |
| QSclav_4359-R | CCCCGCTGTAGATGAGACC | 144 | 62 |
| QSclav_3668-D | GAGCCCGACAACTATCTGGA |  |  |
| QSclav_3668-R | CGTGCGAGACGAAGAGGAC | 91 | 64 |
| Q*mprA2-*D | CAGGCGGTCGTCAAGTC |  |  |
| Q*mprA2-*R | CTATCTGGGTGCGGAAGGT | 109 | 64 |
| QSCLAV_5661-D | CGGCGAACTCTTCAAATGG |  |  |
| QSCLAV_5661-R | GCGTGGGCGAGGATGAC | 108 | 62 |
| Q*hlmA-*D | GGAGTTCATACGGGGTGAGG |  |  |
| Q*hlmA-*R | TACGGGCAGTGGAGCAGA | 195 | 64 |
| Q*hlmI-*D | GCCGTCTCCATCGTCAAC |  |  |
| Q*hlmI-*R | AACCCGTGCGTCTCGTAGT | 178 | 62 |
| Q*ceaS2-*D | GCCGAGCGCCTGAACATCC |  |  |
| Q*ceaS2-*R | GCGGTCCACCGGGGCAACAT | 153 | 60 |
| Q*claR-*D | CGGGCGGCGGTTCTT |  |  |
| Q*claR-*R | TCGTCGAGCAGGGGTTCC | 123 | 60 |
| Q*hrdB-*D | CGCGGCATGCTCTTCCT |  |  |
| Q*hrdB-*R | AGGTGGCGTACGTGGAGAAC | 109 | 60 |
| argR-lux_D | CGCGGATCCATCTCGGAGCATCTGAC |  |  |
| argR-lux_R | CTGCATATGAGACAGCGACGTCTCATTA | 147 | 60 to 55 |
| phoH-lux_D | CGCGGATCCCGCGCTGCGCCGCTACGCCTTCCTGGAC |  |  |
| phoH-lux_R | GCGCATATGTCCCGTTGCAGGGTTCTCG | 277 | 60 to 55 |
| amtB_lux_D | CGCGGATCCCTTCGTCACCCCCGTGAA |  |  |
| amtB_lux_R | GAGCATATGCGACTCCTCGTCGTCGGTGCG | 146 | 60 to 55 |
| Sc_p1123_lux_D | CGCGGATCCTGGCCACGTCGGGGACG |  |  |
| Sc_p1123_lux_R | CCGCATATGCGGAACGAGGCGGCACCAC | 244 | 60 to 55 |
